# Angle- and Polarization-Insensitive Metamaterial Absorber using Via Array

**DOI:** 10.1038/srep39686

**Published:** 2016-12-21

**Authors:** Daecheon Lim, Dongju Lee, Sungjoon Lim

**Affiliations:** 1 School of Electrical and Electronics Engineering, Chung-Ang University, Heukseok-Dong, Dongjak-Gu 156-756, Republic of Korea

## Abstract

In this paper, we propose an angle- and polarization-insensitive metamaterial absorber. We design a metamaterial unit cell that is based on a split ring cross resonator (SRCR). We observe that the absorption frequency and absorption ratio are insensitive to incident angles when a via array surrounds the SRR. We demonstrate the effect of the via array using full-wave simulations by comparing the absorptivity of the SRCR with and without the via array. Because of the symmetric geometry, we also realize polarization insensitivity. We build the proposed absorber on a printed-circuit-board with 30 × 30 unit cells, and we demonstrate its performance experimentally in free space. Under normal incidence, the fabricated absorber shows 99.6% absorptivity at 11.3 GHz for all polarization angles, while for oblique incidence, the fabricated absorber maintains an absorptivity higher than 90% for incident angles up to 70° and 60° for transverse magnetic (TM) and transverse electric (TE) modes, respectively.

Metamaterials are artificial materials with unique features that cannot be found in natural materials^1^. For instance, their permittivity and permeability can be tailored to be negative or highly dispersive. Metamaterials can be realized using the periodic array of resonators such as a split ring resonator (SRR)^2^,^3^,^4^,^5^. Because of their extraordinary features, they have been used for many applications such as compact microwave circuits^6^, super lenses^7^, invisible cloaking^8^, and absorbers^9^.

In particular, microwave metamaterial absorbers are useful for radar cross section (RCS) and electromagnetic interference (EMI) reduction^10^,^11^. Compared with conventional absorbing materials such as ferrite^12^ or composite materials^13^, high absorptivity can be achieved by fabricating metamaterials on low-cost printed circuit boards (PCBs), and an almost perfect absorptivity can be achieved in spite of their thin configuration. However, because metamaterials are based on resonator arrays, their absorptivity is dependent on the frequency and its bandwidth is narrow^14^. Although its narrow bandwidth is useful for sensor applications^15^, a wide bandwidth is preferred for most applications. Therefore, various techniques have been proposed to increase the bandwidth of metamaterial absorbers^16^,^17^,^18^. In addition, its absorptivity is dependent on the polarization and incident angles. Although the polarization insensitivity can be achieved using symmetric unit cells^19^,^20^,^21^, it is difficult to achieve angle insensitivity for metamaterial absorbers. Therefore, many attempts have been made to realize angle-insensitive metamaterial absorbers^22^,^23^,^24^,^25^.

In this paper, we propose a novel angle- and polarization-insensitive metamaterial absorber for X-band applications. We designed its unit cell based on the design of a split ring cross resonator (SRCR)^26^. Because of its symmetric geometry, its absorptivity is the same for all polarization angles; however, its absorptivity varies for different incident angles. In this work, we solve the angle-sensitivity problem by introducing a via array to the outer perimeter of each SRCR. For oblique incidence, the absorptivity of the SRCR with the via array is stabilized compared with the absorptivity of the SRCR without the via array. We demonstrate the proposed idea by performing both full-wave simulations and measurements.

## Principle of metamaterial absorber

Because the permittivity and permeability of metamaterials can be manipulated, perfect absorbers can be realized using metamaterials. High absorptivity can be achieved by minimizing both the reflection coefficient (Γ) and transmission coefficient (T) because the absorptivity (A) is given by


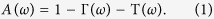


For instance, the absorptivity becomes 100% for reflection and transmission coefficient values of zero.

Under normal incidence, the reflection coefficient is given by


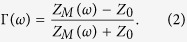


The impedances of the metamaterial (Z_M_) and free space (Z_0_) are given by


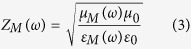



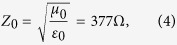


where ε_0_ and μ_0_ are the permittivity and permeability of free space, respectively. In addition, ε_M_ and μ_M_ are the relative permittivity and permeability of a metamaterial, respectively. When Z_M_ is the same as Z_0_, from Eq. (2), we achieve a zero reflection. Therefore, we can achieve a reflection coefficient of zero by tailoring ε_M_ and μ_M_ to be identical each other. When there is no reflected wave, all electromagnetic (EM) energy is transmitted. When the transmitted EM wave is dissipated from dielectric losses, we can achieve zero transmission^27^. Therefore, lossy dielectric materials are preferred for metamaterial absorbers.

However, the zero-reflection condition changes under oblique incidence^28^. For instance, the reflection coefficient for the transverse electric (TE) mode is given by


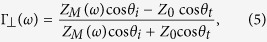


where θ_i_ and θ_t_ are the incident and transmitted angles, respectively. In addition, the reflection coefficient for the transverse magnetic (TM) mode is given by


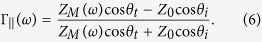


In general, metamaterial absorbers are designed to satisfy a zero-reflection condition under normal incidence. Because the reflection coefficient becomes nonzero under oblique incidence, the absorptivity of a metamaterial absorber is decreased as the incident angle is increased. In order to solve this problem, we propose an incident angle-insensitive unit cell for metamaterial absorbers.

## Unit cell design

**Figure 1:**
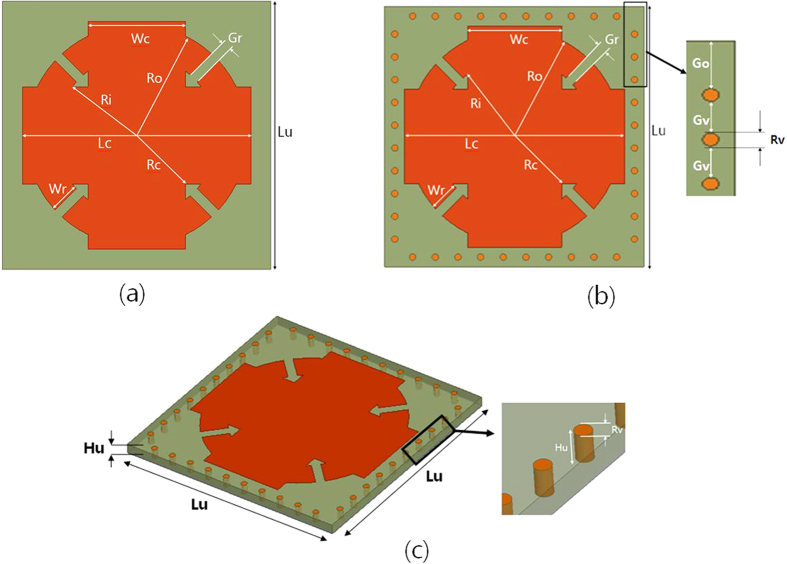
Unit cells of the proposed absorber (**a**) without via array, (**b**) with via array, and (**c**) 3D view of (**b**) (unit: mm) L_u_ = 8, L_c_ = 6.8, R_o_ = 3.3, R_i_ = 2.4, R_c_ = 2.05, W_r_ = 0.9, W_c_ = 2.9, G_c_ = 0.3, R_v_ = 0.2, G_o_ = 0.75, G_v_ = 0.5, and H_u_ = 0.35.

Figure 1 illustrates two unit cells of a metamaterial absorber. The unit cell consists of an SRCR pattern on the top plane and a completely covered ground on the bottom plane. Figure 1(a) shows the primitive SRCR without a via array having geometrical dimensions of L_u_ = 8 mm, L_c_ = 6.8 mm, R_o_ = 3.3 mm, R_i_ = 2.4 mm, R_c_ = 2.05 mm, W_r_ = 0.9 mm, W_c_ = 2.9 mm, and G_c_ = 0.3 mm. Figure 1(b) shows the geometry of the proposed SRCR with the via array. The geometric dimensions of the via array are R_v_ = 0.2 mm, G_v_ = 0.5 mm, and G_o_ = 0.75 mm. Because the unit cell is horizontally and vertically symmetric, its absorptivity is expected to be identical for all polarization angles. Figure 1(c) shows a bird-eye view of the proposed unit cell. In this work, we used an FR-4 substrate with thickness of H_u_ = 0.35 mm. Its relative permittivity and dielectric loss tangent are 4.2 and 0.038, respectively. In general, FR-4 substrates are not used for the X-band because of their high dielectric loss. However, FR-4 substrates are good candidates for absorber applications because of their high dielectric loss and low cost.

We demonstrate the proposed idea by performing a full-wave simulation. In this work, we used a finite-element method (FEM)-based ANSYS high-frequency structure simulator (HFSS). The split ring cross resonator (SCRR) is designed based on LC resonance. Its resonant frequency is calculated by


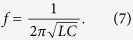


**Figure 2:**
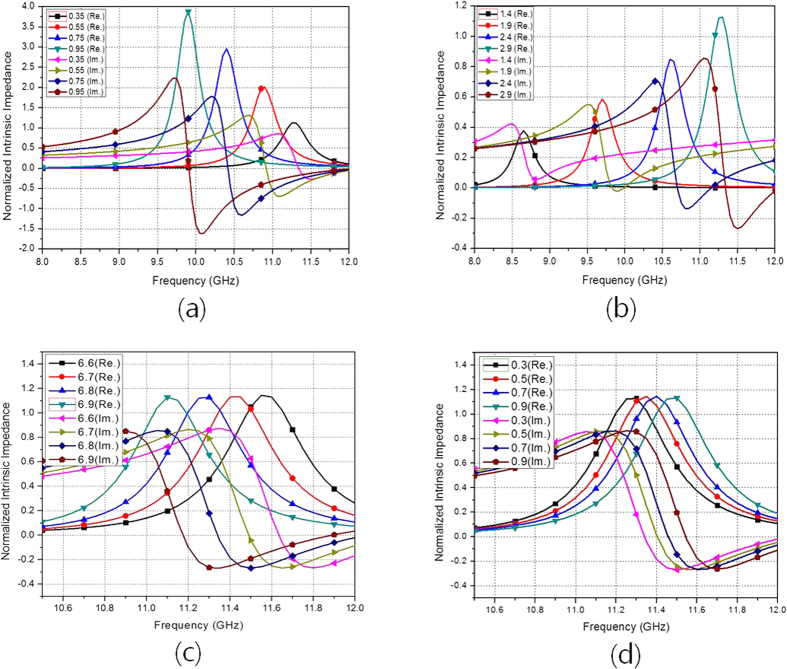
Simulated normalized intrinsic impedances of the proposed metamaterial absorber for (**a**) different H_u_, (**b**) different W_c_, (**c**) different L_c_ and (**d**) different G_r_.

The inductance of the SCRR (L) is mainly determined from the length and width of the cross (L_c_ and W_c_) and thickness of the substrate (H_u_)^29^. The capacitance of the SCRR (C) is mainly determined from the gap spacing (G_r_)^29^. Therefore, as L_c_ and H_u_ increase, the resonant frequency is decreased because of higher inductance. In addition, as W_c_ increases, the resonant frequency is increased because of lower inductance. As G_r_ becomes wider, the resonant frequency increases because of lower capacitance (C). Figure 2 shows impedances of the proposed metamaterial absorber when the geometrical parameters of the SRCR are varied. The thickness of the substrate, H_u_, affects the peak impedance and resonant frequency.

**Figure 3:**
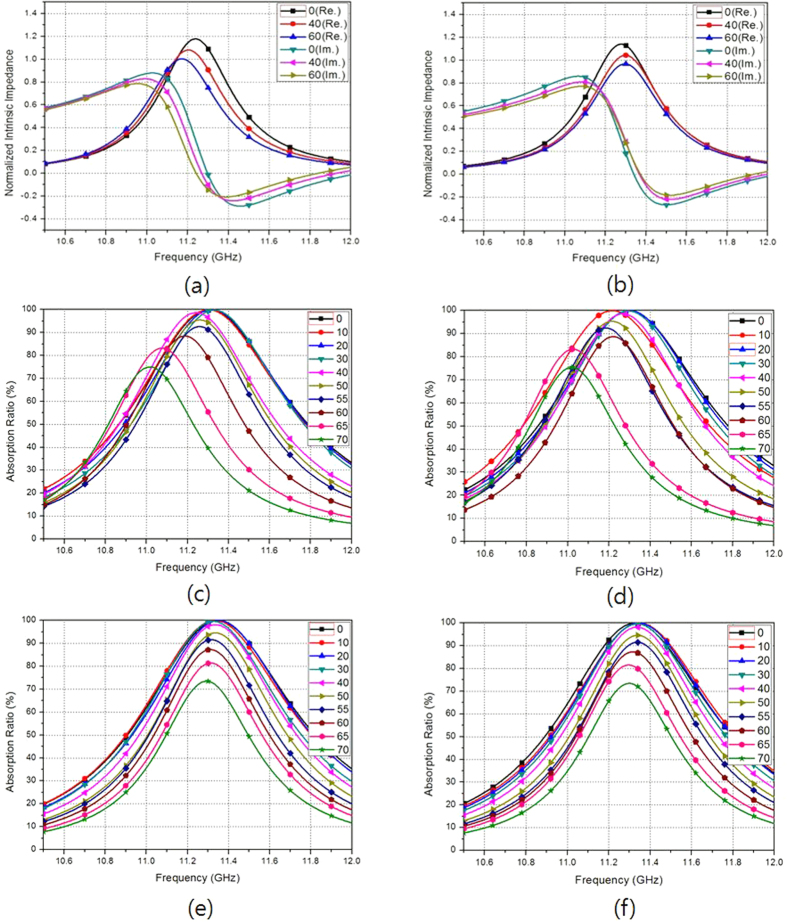
Simulated normalized impedances of the SRCR (**a**) without the via array and (**b**) with the via array for different incident angles. Simulated absorptivity of the SRCR without the via array for (**c**) TE and (**d**) TM modes. Simulated absorptivity of the SRCR with the via array for (**e**) TE and (**f**) TM modes.

**Figure 4:**
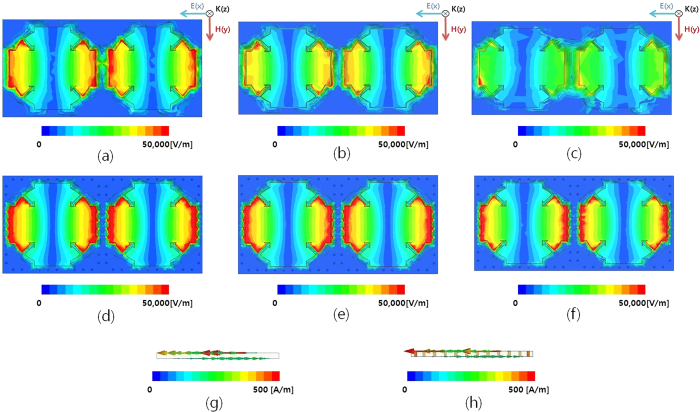
Magnitude of electric-field distribution of the SRCR without the via array: (**a**) under normal incidence array (*θ* = 0°) and under oblique incidence ((**b**) *θ* = 40° and (**c**) *θ* = 60°). Magnitude of electric-field distribution of the SRCR with the via array: **(d**) under normal incidence array (*θ* = 0°) and under oblique incidence ((**e**) *θ* = 40° and (**f**) *θ* = 60°). Vector surface current densities of the SRCR (**g**) without and (**h**) with the via array under normal incidence.

It is observed from Fig. 2(a) shows the resonant frequency is decreased and peak impedance is increased when H_u_ is increased from 0.35 mm to 0.95 mm. The width of the cross W_c_ affects the peak impedance and resonant frequency. It is observed from Fig. 2(b) shows the resonant frequency and peak impedance are increased when W_c_ is increased from 1.4 mm to 2.9 mm. In addition, Fig. 2(c) shows the resonant frequency is decreased when L_c_ is increased from 6.6 mm to 6.9 mm while the peak impedance is almost not changed. It is observed from Fig. 2(d) shows that the resonant frequency is increased when G_r_ is increased from 0.3 mm to 0.9 mm. Finally, its parameters are determined as L_u_ = 8, L_c_ = 6.8, R_o_ = 3.3, R_i_ = 2.4, R_c_ = 2.05, W_r_ = 0.9, W_c_ = 2.9, and G_c_ = 0.3 [unit: mm]. Figure 3(a) and (b) show the simulated impedances of the SRCR without and with the via array for different incident angles. There is no difference in the intrinsic impedance with and without the via under normal incidence. It is expected that the resonant frequency of the SRCR without the via array will be 11.31 GHz, while the resonant frequency of the SRCR with the via array is 11.35 GHz under normal incidence. However, when the incidence angle is wider, less electric field at the edges of the SCRR without the via array is coupled to the edges of adjacent SCRRs. It is observed from the electric field distribution in Fig. 4(a,b,c). Difference of coupling level between unit cells result in variations of frequency and impedance. Therefore, the via array is introduced to eliminate mutual coupling between adjacent unit cells. In order to reduce fabrication cost of vias, 0.5 mm via spacing (G_v_) are used with 0.2 mm diameters (R_v_) of via holes. Although the minimum G_v_ is 0.15 mm due to fabrication limitation, the absorptivity is not changed from up to 0.5 mm. After loading the via array with 0.5 mm of G_v_ and 0.2 mm of R_v_, it is observed from Fig. 4(d,e,f) that electric fields at the edges of the SCRR are not coupled to the edges of adjacent SCRRs and their magnitudes are not changed although the incident angle changes. Figure 3(c,d,e,f) show the simulated absorptivity of the SRCR for the TE mode and TM mode with and without the via array, respectively, under oblique incidence. Because the bottom plane is completely covered by the conductor, the transmission coefficient must be zero, and the absorptivity is determined from only the reflection coefficient in Eq. (1). We clearly observed that the SRCR with the via array shows more stable absorptivity compared with the SRCR without the via array. For a quantitative comparison, the angle-sensitivity (S_A_) is defined as^30^,^31^


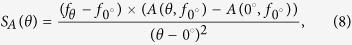


where *f*_θ_ is the resonant frequency at the incident angle of *θ*, and A(*θ, f*_0°_) is the absorptivity at the incident angle of *θ* and the resonant frequency under normal incidence (*f*_0°_). Therefore, both frequency and absorptivity variations are considered in S_A_. For each incident angle, Table 1 shows a comparison of the peak absorption frequencies and S_A_ of the SRCR with and without the via array. For the primitive SRCR of Fig. 1(a), the resonant frequency and absorptivity decreases when *θ* increases. For the proposed SRCR of Fig. 1(b), the resonant frequency does not change until *θ* is 70°, and the absorptivity is maintained higher than 90% until 55° for the TM and TE modes, respectively. For instance, the angle sensitivity (S_A_) with the via array is 3.57 × 10^−4^ at *θ* = 70°, while the S_A_ without the via array is 3.64 × 10^−3^ at *θ* = 70°.

The electric and magnetic resonance of the proposed metamaterial absorber can be observed from electric-field distribution and electric current densities^26^. Figure 4(a) and (b) show the simulated magnitude of the electric field distributions of the SRCR without and with the via array under normal incidence, respectively. The electric resonance is generated from strong capacitive coupling at the edge and gap. Figure 4(c) and (d) show the simulated magnitude of the electric-field distributions of the SRCR without and with the via array under oblique incidence (*θ* = 40°), and Fig. 4(e) and (f) show the simulated magnitude of the electric-field distributions of the SRCR without and with the via array under oblique incidence (*θ* = 60°) respectively. Figure 4(g) and (h) show vector surface current densities of the SRCR without and with the via array under normal incidence, respectively. The magnetic resonance is generated from the surface current on the top and bottom planes, which are anti-parallel to each other.

## Fabrication and measurement results

**Figure 5:**
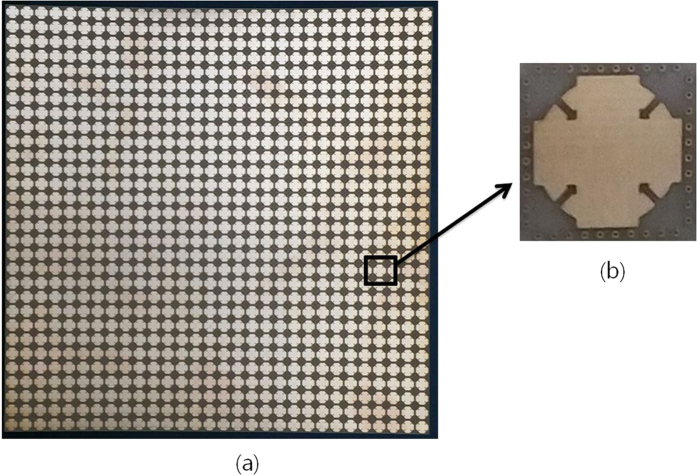
Pictures of (**a**) fabricated metamaterial absorber prototype and (**b**) enlarged view of a unit cell.

In order to demonstrate the performances of the proposed metamaterial absorber, the prototype with 30 × 30 unit cells are fabricated on an FR-4 substrate. Figure 5 shows a picture of the fabricated prototype with an overall size of 240 mm × 240 mm. We used copper for the conductive patterns on the top and bottom planes. The via array is realized by filling copper into the via holes after they are drilled.

**Figure 6:**
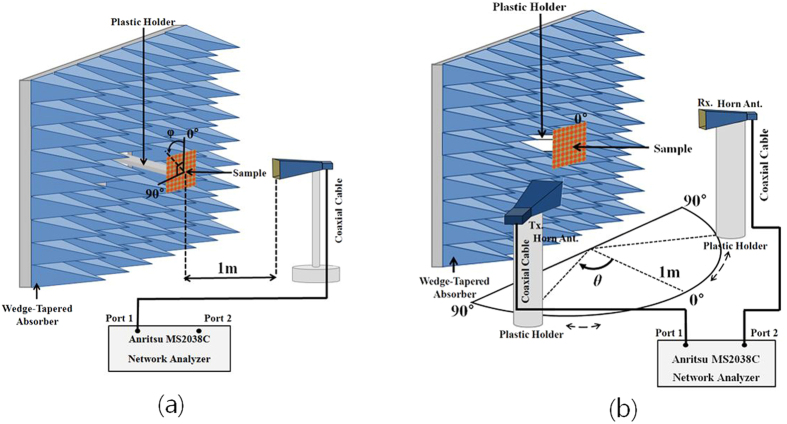
Illustration of free-space measurement setup for (**a**) normal incidence and (**b**) oblique incidence.

**Figure 7:**
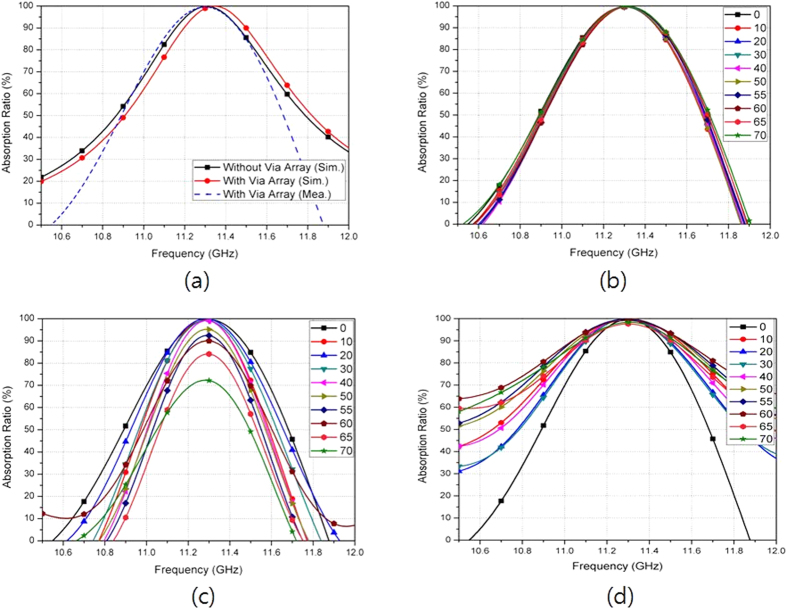
(**a**) Simulated and measured absorptivity of the metamaterial absorber with and without the via array under normal incidence. (**b**) Measured absorptivity of the fabricated absorber sample for different polarization angles (φ) under normal incidence. Measured absorptivity of the fabricated absorber sample for different incident angles (*θ*) under (**c**) TE mode and (**d**) TM mode.

We measured the absorptivity of the fabricated prototype in free space. Figure 6 illustrates the measurement setup. We used an Anritsu MS2038C vector network analyzer (VNA) to measure the S-parameters. From Eq. (1), we calculated the absorptivity from the reflection coefficient and transmission coefficient. The reflection coefficient is calculated from the S-parameters and the transmission coefficient is zero because of the completely conductive bottom plane. We used the single horn antenna to measure the absorptivity under normal incidence. Then, we compared the measured absorptivity of the fabricated metamaterial absorber with its simulated absorptivity in Fig. 7(a). We observed that the measured absorptivity is 99.6% at 11.3 GHz, while the simulated absorptivity is 99.99% at 11.35 GHz. Therefore, the simulated and measured results show good agreement around the resonant frequency. As the frequency is farther away from the resonant frequency, difference between the simulation and measurement results becomes larger because we used constant permittivity and tangential loss to characterize the FR-4 substrate in EM simulation. Its permittivity and tangential loss are dependent on a frequency.

From the measurement result, we achieved an absorptivity of higher than 90% from 11.14–11.46 GHz. In addition, from the simulation results, we observed that the absorptivity remains almost constant although the via array is loaded on the SRCR. The simulated resonant frequency of the SRCR with the via array is 11.35 GHz, while the simulated resonant frequency of the SRCR without the via array is 11.31 GHz.

In order to see the polarization sensitivity of the proposed absorber, the horn antenna is rotated for a polarization angle (*φ*) ranging from 0°–90° under normal incidence. Figure 7(b) shows the measured absorptivity of the fabricated prototype at different polarization angles. As expected, the absorptivity is not changed because the unit cell is both vertically and horizontally symmetric.

We used two horn antennas to measure the absorptivity under oblique incidence. One antenna is used to transmit EM energy, and its incident angle is changed by rotating the horn antenna from 0° to 80°. The other horn antenna is used to receive the EM energy reflected from the absorber, and it is placed at an angle to satisfy Snell’s law. Figure 7(c) and (d) show the measured absorptivity of the fabricated prototype at the TE and TM modes, respectively. For the TM mode, the absorptivity and resonant frequency remain unchanged up to *θ* = 60°, while keeping an absorptivity of almost 99% at 11.3 GHz. In addition, the absorptivity is higher than 95% although the incident angle is varied up to 70°. For the TE mode, the absorptivity decreases as the incident angle increases. However, an absorptivity greater than 90% is kept up to *θ* = 60° and the peak absorption frequency is varied from 11.27 GHz to 11.33 GHz. The absorptivity at *θ* = 70° is 70% with a peak absorption frequency of 11.28 GHz. Therefore, by introducing the via array to the outer perimeter of the SRCR unit cell, from the measurement results obtained, we successfully demonstrated that the absorptivity of the proposed metamaterial absorber is insensitive to the incident angle.

## Discussion

In this paper, we proposed a novel angle- and polarization-insensitive metamaterial absorber for use in the X-band. The unit cell of the proposed absorber is initiated from the SRCR, which is insensitive to polarization angles because of its vertical and horizontal symmetry. However, the absorptivity of the SRCR unit cell is sensitive to incident angles. When the via array is loaded to the outer perimeter of the SRCR, the absorptivity and peak absorption frequency become insensitive to incident angles. In this work, we investigated the effect of the proposed via array from full-wave simulations by comparing the absorptivity without and with the via array. The angle sensitivity (S_A_) with the via array is 3.57 × 10^−4^ at *θ* = 70°, while the S_A_ without the via array is 3.64 × 10^−3^ at *θ* = 70°. We demonstrated the proposed idea from free-space measurements performed after fabricating 30 × 30 unit cells, which corresponds to 240 mm × 240 mm. Under normal incidence, the absorptivity is 99.6% at 11.3 GHz for all polarization angles. Under oblique incidence, the absorptivity at the TM mode remains higher than 95% up to *θ* = 70°. The absorptivity at the TE mode remains higher than 90% up to *θ* = 60°. Therefore, both angle- and polarization-insensitivity of the proposed metamaterial absorber is successfully demonstrated from full-wave simulations and measurements.

In Table 2, we compared the absorptivity of the proposed metamaterial absorber with the absorptivity of other angle-insensitive metamaterial absorbers. The absorption ratio of the proposed metamaterial absorber is higher than other angle-insensitive metamaterial absorbers for incident angles of up to 70°.

## Methods

### Simulation

In order to simulate performances of the proposed absorber, we used a finite-element method (FEM)-based ANSYS high-frequency structure simulator (HFSS). The proposed metamaterial absorber is a periodic structure. In order to set a unit cell with an infinite periodic array, we assigned two “Master” and “Slave” pairs on the surfaces as boundary conditions in the ANSYS HFSS setup. In addition, we used a Floquet port to excite EM energy to the unit cell, and we calculated the absorptivity from the S-parameters. Because the polarization angle (*φ*) and incident angle (*θ*) are defined as “phi” and “theta” on the Floquet port, we simulated the absorptivity for each *φ* and *θ* by varying “phi” and “theta” on the Floquet port. We also plotted the magnitudes of the electric fields and vector electric current densities using ANSYS HFSS.

### Measurement

We measured the absorptivity of the fabricated metamaterial absorber in free space, as illustrated in Fig. 6. We calculated the absorptivity from the S-parameters that were measured using an Anritsu MS2038C VNA. We used a single horn antenna to measure S_11_ under normal incidence, as shown in Fig. 6(a). We used two horn antennas to measure S_21_ under oblique incidence, as shown in Fig. 6(b). The absorber prototype is located 1 m away from the horn antennas in order to satisfy the far-field condition. In addition, the absorber prototype is surrounded by wedge-tapered absorbing materials to remove unwanted reflected and scattered EM waves. In order to receive the reflected EM wave only from the absorber prototype, we applied a time-gating function of the VNA. Before measuring the S-parameters of the absorber prototype, we first measured the S-parameter of the copper plate and set the magnitude of its reflection coefficient to be 1 for the calibration process. In order to measure the absorptivity at different polarization angles, we rotated the horn antenna from 0° to 90°, while fixing its location at *θ* = 0°, and we measured the S-parameters at each polarization angle. In order to measure the absorptivity at different incident angles, the transmitting horn antenna is rotated from 10° to 70° on the azimuth plane. At each incident angle of *θ*, we placed the receiving horn antenna at that angle to satisfy Snell’s law, and we measured the S-parameters at each incident angle.

## Additional Information

**How to cite this article**: Lim, D. *et al*. Angle- and Polarization-Insensitive Metamaterial Absorber using Via Array. *Sci. Rep.* **6**, 39686; doi: 10.1038/srep39686 (2016).

**Publisher's note:** Springer Nature remains neutral with regard to jurisdictional claims in published maps and institutional affiliations.

## Figures and Tables

**Table 1:** Peak absorption frequency and angle-sensitivity (S_A_) of the SRCR without and with the via array when the incident angle is varied from 0 to 70°.

	*θ* [degree]	10	20	30	40	45	50	55	60	65	70
Without Via Array	*f*_θ_ [GHz]	11.3	11.32	11.33	11.24	11.33	11.26	11.26	11.19	11.08	11.02
	S_A_(*θ*) × 10^−6^	4.142	2.970	6.930	5.197	0.028	122.2	152.8	741.2	2509	3638
With Via Array	*f*_θ_ [GHz]	11.33	11.35	11.32	11.34	11.35	11.34	11.32	11.31	11.31	11.29
	S_A_(*θ*) × 10^−6^	16.70	0	25.51	0.236	0	21.96	91.41	154.2	185.8	357.1

**Table 2:** Compared performances of the proposed metamaterial absorber with other angle-insensitive metamaterial absorbers.

	Frequency	A at *θ* = 0°		A at *θ* = 50°	A at *θ* = 60°	A at *θ* = 70°	Polarization Insensitivity
Ref. 19	10.14 GHz	97%	≈	99%	92%	None	Yes
Ref. 20	0.22 THz	97%		97%	97%	None	Yes
Ref. 21	9 GHz	97%		—	93.72%	None	No
Ref. 22	10.3 GHz	97%		99%	95%	None	Yes
Ref. 26	10.44 GHz	99%		97%	90%	80%	Yes
Proposed Work	11.3 GHz	99%		99%	99%	98%	Yes
